# Barriers to Population Data Use in Overdose Fatality Reviews: Observational and Interview Study of Dashboard Deployment

**DOI:** 10.2196/98864

**Published:** 2026-07-27

**Authors:** Amey Salvi, Cynthia Holladay, Allyson L Dir, Bradley Ray, Matthew C Aalsma, Khairi Reda

**Affiliations:** 1 School of Informatics, Computing, and Engineering, Indiana University Indianapolis, Indianapolis, IN, United States; 2 Department of Pediatrics, Indiana University School of Medicine, Indianapolis, IN, United States; 3 Department of Psychiatry, Indiana University School of Medicine, Indianapolis, IN, United States; 4 RTI International, Research Triangle Park, NC, United States; 5 Department of Pediatrics, Children’s Health Services Research, Indiana University School of Medicine, Indianapolis, IN, United States; 6 Department of Computer Science, University of Illinois Chicago, 850 W Taylor St, CDRLC 5409, Chicago, IL, 60607, United States, 1 3123550271

**Keywords:** dashboard, visualization, public health, overdose, overdose prevention, recommendations, observation study, fatality review, prevention, interview, touchpoints, substance abuse

## Abstract

**Background:**

Overdose fatality reviews (OFRs) are an important public health tool for developing local overdose prevention strategies by reviewing individual overdose cases. While this approach offers a rich, contextual understanding of drug overdose factors in communities, it examines a small number of cases, providing limited insight into broader population-level risk patterns. To complement OFRs, we developed a real-time dashboard that visualizes trends about 5 key “touchpoints” (ie, interactions with medical and justice services preceding overdose). We then trained local OFR teams to use this dashboard to identify prevention opportunities.

**Objective:**

This study examines the integration of population-level data into OFR practices, as well as the tensions that emerge between OFRs’ traditional case-driven review processes and the statistical, population-level analysis typical in public health. We analyze how and when the dashboard was used in meetings, determine the extent to which the data informed recommendations, and identify barriers that prevented the broad uptake of this intervention.

**Methods:**

We observed 26 OFR meetings across 11 counties in Indiana over 10 months, from November 2024 to September 2025, during which teams conducted case reviews and developed recommendations. We documented instances of dashboard use as well as “missed opportunities,” in which relevant population-level data were available but not incorporated into the discussion. We also conducted semistructured interviews with OFR team members (n=7) to understand their perceptions of the dashboard, including its usefulness, usability, and adoption barriers.

**Results:**

Despite its intended role, the dashboard was rarely integrated into OFR meetings; it was used only 10 times, compared to 114 missed opportunities in which relevant data could have informed discussions. Interviews revealed that this limited uptake was not solely due to usability barriers but reflected a deeper tension between 2 distinct analytic approaches. OFR teams prioritized narrative-driven case reviews that were grounded in empathy, local knowledge, and lived experience. This approach seemed at odds with the population-level visualizations shown in the dashboard, which required statistical abstraction and interpretation. Other barriers identified included limited time and resources, staff turnover, and varying levels of data fluency, which made it difficult for teams to confidently interpret the dashboard despite training.

**Conclusions:**

The results highlight a tension between case-based and data-driven approaches to overdose prevention. These approaches are grounded in different workflows, values, and motivations, making it challenging for OFR teams to maintain their traditional, empathetic review practices while incorporating population-level trends. Our findings indicate the need for data tools that bridge these approaches, such as visualizations that connect aggregate patterns to individual cases. The results also underscore the need for additional support and training for teams, such as dedicated data specialists who interpret population-level trends and provide insights to augment team discussions and inform prevention strategies.

## Introduction

The United States has seen a dramatic rise in drug overdoses over the past two decades, from 6.1 to 32.4 deaths per 100,000 people between 2001 and 2021 [1]. Although overdose fatalities have declined recently, the number remains high [2,3]. The response to this epidemic is further complicated by variations in risk factors across regions [4] and subpopulations [5]. Addressing this crisis thus requires data- and evidence-informed interventions tailored to the needs and challenges of local communities.

To encourage effective local overdose prevention, the Centers for Disease Control and Prevention has promoted local overdose fatality review (OFR) teams, first implemented in Maryland in early 2014 [6]. These teams unite public health, law enforcement, and emergency response stakeholders to review overdose cases. By analyzing decedents’ histories and their interactions with services, teams can identify risks and gaps and recommend targeted prevention strategies [7]. Although some OFR teams have generated effective recommendations [6], this review model typically draws insights from only a handful of cases (2‐3 per month or quarter) [8]. Such reviews provide rich qualitative insights into system and service gaps in local communities but may lead to recommendations that overlook broader overdose risk factors. Although some teams supplement their case reviews with quantitative data, these analyses are often based on small sample sizes, offering only a partial picture. This limitation presents an opportunity to enhance the OFR model by incorporating population-level data through a real-time dashboard, enabling more comprehensive, data-driven recommendations.

To address this opportunity, we partnered with the Indiana state government starting in September 2022 to develop and deploy a dashboard that provides OFR teams with real-time data about touchpoints—events and services individuals interact with *before* experiencing a fatal overdose [9,10]. The dashboard provides statistics about key touchpoints, including interactions with emergency and medical services and justice systems [11]. To introduce local OFR teams to the dashboard and provide guidance on using its data, we invited participating teams to a 2-day training workshop focused on population-level data use and evidence-based overdose prevention.

While dashboards have found success in public health settings [12-15] and are extensively used to monitor overdose trends [16-18], their use by OFR teams has received little attention. Even when team members receive training on integrating the dashboard, it represents a new, potentially fundamental change to the OFR process. Teams can also be highly diverse, including many stakeholders with no formal training or experience in data analysis. Thus, it is important to understand how data-driven approaches to overdose prevention are adopted within OFRs, including potential barriers to their uptake.

The goal of this study is to evaluate how well OFR teams can integrate a population-level touchpoints dashboard into their existing practices and whether they can inform their recommendations using touchpoint data alongside traditional case reviews. Specifically, we ask: (1) How frequently do teams consult the dashboard during OFR meetings? (2) To what extent do team members draw insights from dashboard data to make actionable recommendations for preventing overdoses? (3) What challenges do OFR teams face in effectively incorporating the dashboard into their existing workflows? To answer these questions, we observed OFR team meetings and analyzed their dashboard use, as well as any missed opportunities. We then followed up with interviews with team members to better understand their perceptions of the dashboard’s utility and the barriers they faced in fully using it.

## Methods

### Study Setting

The study took place in the state of Indiana as part of the FORTRESS (Fatal Overdose Review Teams: Research to Enhance Surveillance Systems) project. Indiana has encouraged and expanded the use of local OFR teams in recent years, with teams organized at the county level. Most teams convene to review cases in a single county, but some teams oversee multiple jurisdictions, particularly in rural, low-population areas of the state. Organizational support and resources for OFR teams are provided by the Indiana Department of Health (IDOH).

**Figure 1.**
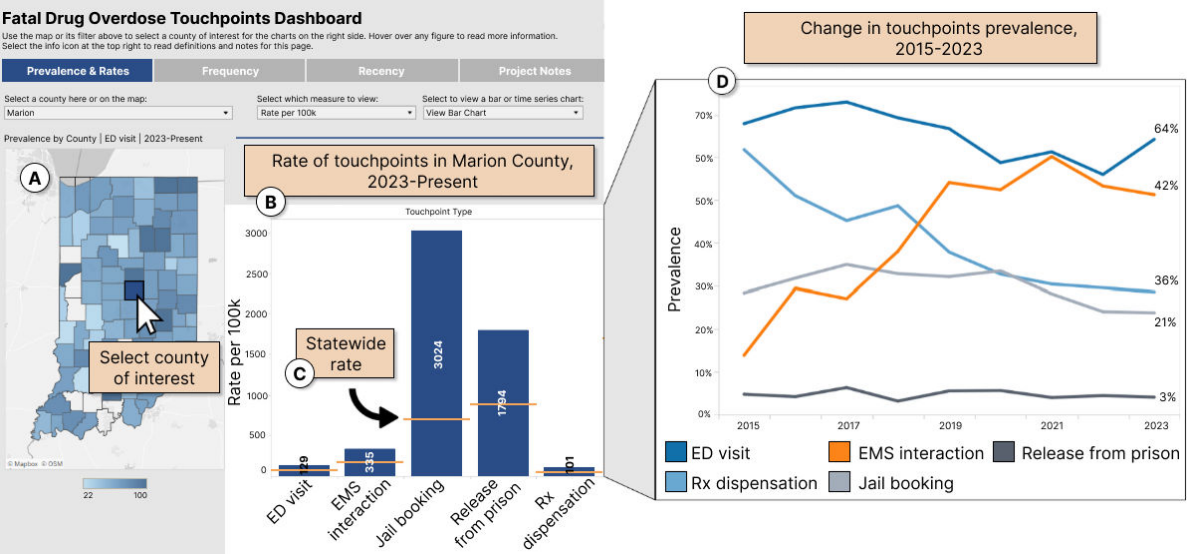
The FORTRESS (Fatal Overdose Review Teams: Research to Enhance Surveillance System) dashboard [19] shows overdose touchpoints in Indiana at the county level. The dashboard provides data on 5 touchpoints: interactions with the emergency health services (ED and EMS), legal systems (jails and prisons), and the dispensation of scheduled drugs. Visualizations illustrate the spatial distribution of touchpoints in the state (A), the rate of touchpoints in the selected county (B) versus the state average (C). The dashboard can also show how the prevalence and rates of touchpoints have changed over time, both within counties and across the whole state (D). ED: emergency department; EMS: emergency medical services; Rx: medical prescription.

The touchpoints dashboard used in this study was designed and developed through a user-centered design process that included formative focus groups with OFR experts to identify data needs, followed by an iterative design and feedback process. The expert group included experienced OFR practitioners as well as early developers of the OFR model from across the United States. The dashboard uses a record-linking process, integrating state mortality and administrative datasets to assemble a history of decedents’ interactions with touchpoints and covers all overdose fatalities in the state between 2015 and the present. The focus on visualizing touchpoints that precede overdoses, as opposed to showing overdose trends [16-18], is meant to encourage a prevention-oriented mindset, consistent with the goal of OFRs. Five touchpoints are included in the dashboard: visits to the emergency department (ED), encounters with emergency medical services (EMS), dispensation of prescription drugs (Rx), jail bookings, and releases from prison. The dashboard supplies aggregate statistics displaying decedent data in the 12 months preceding their fatal overdose. These include the prevalence of these touchpoints (eg, percentage of decedents who had visited the ED in the year preceding their fatal overdose), and the rate of overdose among all touchpoint users (eg, the fraction of prison releasees who went on to experience a fatal overdose in the 12 months following their release). These two metrics help teams estimate both the potential reach of interventions in their community (prevalence) and the relative risk associated with specific touchpoints (rate). Additional measures provided by the dashboard include touchpoint frequency (average number of encounters) and the mean time between the last touchpoint interaction and death. Crucially, the dashboard, first deployed in 2023, breaks down all statistics by county, allowing teams to view data in their locality. Figure 1 illustrates two views from the dashboard. Full details can be found in Salvi et al [11]. The dashboard data are continuously refreshed in (near) real time, approximately every 1 to 2 weeks for most touchpoints.

### Recruitment and Implementation Strategy

We recruited OFR teams for the study by reaching out to all active teams within the state. The list of active teams and their contacts was provided by IDOH. At the start of recruitment in February 2022, Indiana had nearly 2 dozen active OFR teams, with 5 more teams expected to begin meeting within the year. Of these teams, 19 responded to our recruitment and provided consent and letters of support to participate in the study. The recruited teams represented a variety of counties within the state, including both rural and urban communities. Recruited OFR teams were organized and introduced to the study using a stepped-wedge cluster design [20], in which 3 cohorts received training in successive waves (in October 2024, May 2025, and October 2025). Cohort 1 included 6 OFR teams, Cohort 2 included 8, and Cohort 3 included 5. Each training session lasted 2 days and covered dashboard functionality and data interpretation, evidence-based prevention practices, and strategies for developing actionable recommendations. Two members from each team, including the facilitator, were invited to participate in the training.

This study includes only observational and interview data from Cohorts 1 and 2, as Cohort 3 had not yet been observed at the time of writing. We sampled 11 OFR teams from the first 2 cohorts: 5 from Cohort 1 and 6 from Cohort 2. Teams were selected based on meeting schedules and the observer’s availability. The observer attempted to maximize the number of meetings observed by attending as many meetings as feasible within the study period. All 11 teams observed had completed the 2-day training. Observations for Cohort 1 teams were conducted between November 2024 and September 2025, and for Cohort 2 teams between July 2025 and September 2025. Following observations, we recruited 7 OFR team members for one-on-one interviews via email. We identified prospective interviewees through OFR team mailing lists and sent recruitment messages that included the purpose, duration, compensation, and a formal information sheet. We reached out to 9 members, 7 of whom ultimately responded and agreed to participate in the interview. Recruitment prioritized facilitators, given their depth of experience with the OFR process and their key role in conducting the meetings, although participation was not limited to them.

### Data Collection

Meetings primarily covered overdose cases, with some also covering suicide cases (which we did not analyze), lasting 2 hours on average. A member of the research team (the first author) attended all meetings as an observer and documented the discussions by taking notes on dashboard use and missed opportunities. The same researcher then conducted semistructured follow-up interviews with 7 members from different OFR teams. Interviews represented diverse professional backgrounds and varying levels of experience with data. Interviewees included a registered nurse (RN), a police social worker (PSW), a nonprofit organization director, a behavioral health data analyst, a medication-assisted treatment (MAT) specialist, and OFR facilitators (see Table 1). The interviews were conducted over Zoom and lasted 20 to 30 minutes. Interview questions focused on participants’ experiences with data, their perceptions of dashboard usability and utility, and the barriers they faced to its use (see Multimedia Appendix 1). This paper reports on insights from both observational studies and interviews [21,22]. This combination has been proven useful [23-25], particularly for evaluating dashboard use and utility for making data-driven decisions [26].

**Table 1.** Summary of interview participants’ backgrounds and data experience.

Participant ID	Role/background	Extent of data experience	Served as OFR^a^ facilitator?
P1	Nonprofit organization director	Research assistant with data analysis experience during undergraduate and graduate studies	Yes
P2	Project manager	No data tool training apart from FORTRESS^b^	No
P3	Behavioral health data analyst	No data tool training apart from FORTRESS	Yes
P4	OD2A^c^ contractor	Previous experience with similar dashboards	Yes
P5	MAT^d^ specialist	OFR training in addition to FORTRESS training	Yes
P6	Registered nurse	Graduate degree in statistics	Yes
P7	Police social worker	Extensive experience in data (collection and analysis)	Yes

a OFR: overdose fatality review.

b FORTRESS: Fatal Overdose Review Teams: Research to Enhance Surveillance System.

c OD2A: Overdose Data to Action.

d MAT: medication-assisted treatment.

### Data Analysis

During the observations, we recorded instances in which the team’s discussions and case reviews referenced 1 of the 5 touchpoints on the dashboard. Specifically, we noted: (1) dashboard use, where teams consulted the dashboard, for example, by looking up the prevalence or rates of touchpoints, (2) missed opportunities, where the discussion revolved around touchpoints for which there were data in the dashboard but where teams did not make an attempt to consult those statistics, and (3) nondashboard touchpoints (eg, housing-related) that the team discussed but for which there were no quantitative data. In addition to case discussions, we also analyzed the team’s recommendations, focusing on dashboard use and missed opportunities. To analyze meeting data, we developed a codebook through an iterative coding process. We started with an initial set of codes that covered touchpoints referenced during the meetings, dashboard uses, and recommendations made. The codebook was refined iteratively and expanded to identify more specific types of dashboard use and missed opportunities. Meeting notes and observations were entered into an Excel sheet and assigned appropriate codes.

For the interview data, we used thematic analysis to identify recurring themes [27]. The interviews were video-recorded and transcribed. Interview questions suggested preliminary thematic categories related to dashboard usability, how it complements case reviews, challenges, and future improvements. We supplemented these with an open coding process to identify and tag additional topics that emerged from participants’ responses. We then iteratively refined the codes, developed a codebook, and grouped the final codes into 3 broad themes relating to (1) the extent of dashboard use during and outside meetings, (2) advantages of the dashboard, and (3) challenges in using and interpreting the dashboard. See Checklist 1 for the completed Consolidated Criteria for Reporting Qualitative Research (COREQ) checklist for the study design and analysis.

### Ethical Considerations

This study was reviewed and approved by the Indiana University Institutional Review Board (15922). Participants in the interviews received an information sheet explaining the study goals and procedures before agreeing to take part. Participants received a US $40 gift card as compensation for the interviews. The dashboard displays only aggregate, population-level visualizations. No individual records were displayed in the dashboard to preserve anonymity.

## Results

### Dashboard Usage and Missed Opportunities

We first present our observations from OFR meetings on dashboard use and nonuse. Each OFR meeting typically lasted 2 hours, during which teams discussed 2 to 3 decedent cases. We observed a total of 48 overdose cases being discussed. The case reviews covered decedents’ demographic backgrounds, obituaries, toxicology reports, medical histories, prescriptions for controlled substances and other medications, justice involvement, and next-of-kin interviews. Based on these, a timeline was constructed for each decedent that highlights the major events and touchpoints in their lives. The team discussed the case, identified service gaps, and recommended programmatic changes or policies to prevent future overdoses. Out of the 26 meetings we observed, the dashboard was used in only 2 meetings, where it was referenced either during case reviews or for making recommendations. Some meetings (10/26, 38.46%) also included a discussion of the county’s overdose trends and counts, typically using historical data from prior years obtained from the coroner’s office. However, none of this data overlapped with or provided detailed touchpoint metrics like those available in the FORTRESS dashboard.

**Figure 2.**
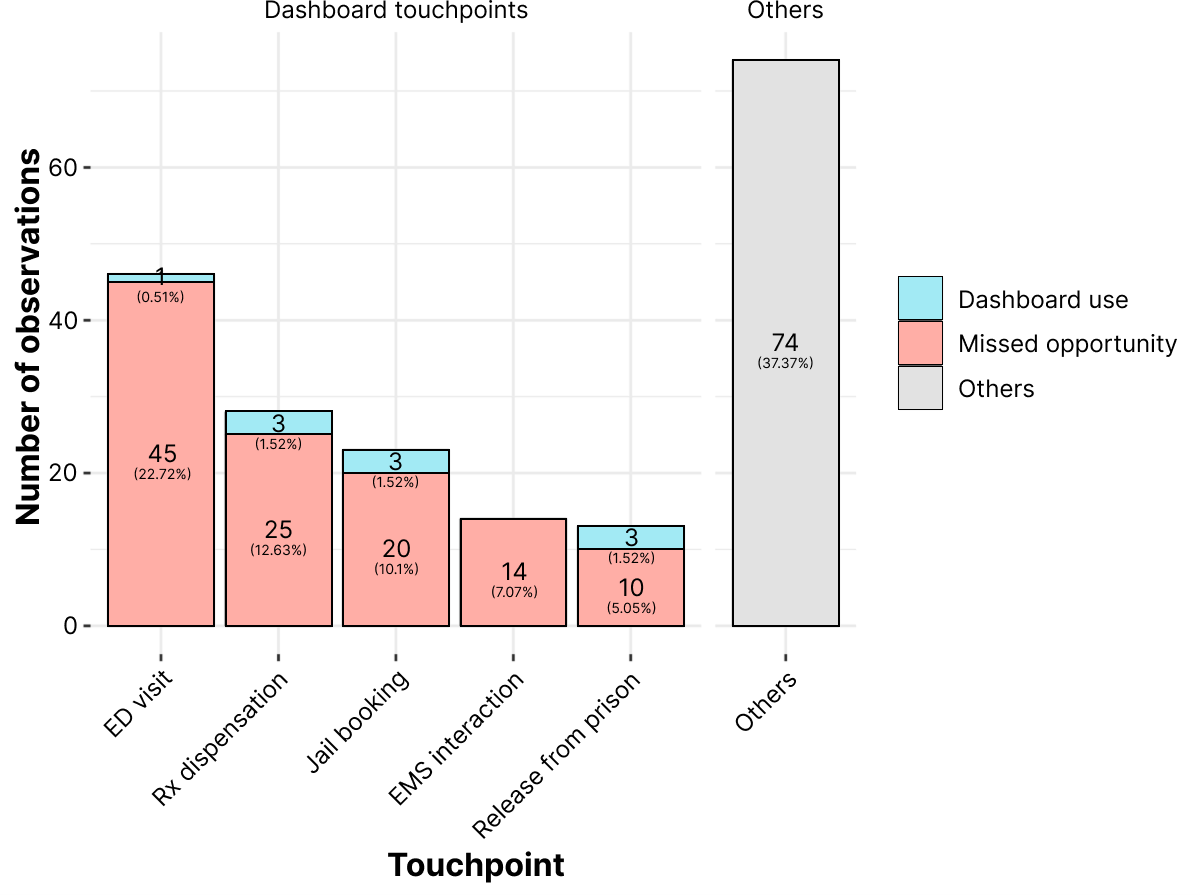
Frequency of touchpoints from all observed meetings. ED: emergency department; EMS: emergency medical services; Rx: medical prescription.

To identify where the dashboard had been—or could have been—used, we coded all touchpoint occurrences in cases where the dashboard provides data. Recall that the dashboard provides data about 5 touchpoints. The most frequently occurring touchpoint was an ED visit, which occurred in 37.1% (46/124) of cases. This was followed by Rx dispensation (28/124, 22.58%), jail bookings (23/124, 18.55%), EMS interactions (14/124, 11.29%), and releases from prison (13/124, 10.48%). In addition, we identified 74 other touchpoints (ie, interactions with systems such as mental health support, temporary housing, and system enhancements) for which the dashboard does not provide data. When OFR teams encountered 1 of the 5 touchpoints in a case, they used the dashboard 10 times across all observed meetings. By contrast, we identified 114 missed opportunities in which cases included touchpoints for which the dashboard provides data, but the information was not looked up or used during the discussion. Figure 2 illustrates the frequency of data use and missed opportunities across the 5 touchpoints.

Although limited, the use of the dashboard during meetings ranged from simple data readouts to contextual interpretation of the data and, in some cases, to generating actionable recommendations. At a basic level, there were 2 instances in which teams simply read data aloud when a case included 1 of the 5 touchpoints. For example, in one meeting, the facilitator distributed printouts showing the prevalence and rate of fatal overdoses associated with the ED touchpoint, noting that their county had a higher prevalence (73% of decedents had visited the ED in the year prior to death, compared to 64% statewide). This data, however, were not taken up further in the discussion. In a more interpretive use pattern, there were 4 instances in which teams engaged with the data by drawing on local knowledge to make sense of population-level trends in their community. For example, in one meeting, the team noted that individuals released from prison had a higher rate of fatal overdose in their county (1067 per 100,000 releases, compared to 704 statewide). This prompted a discussion of this elevated risk, with team members attributing it to factors such as lack of housing, limited support services, and barriers like suspended driver’s licenses upon release. At the highest level of use, we found 4 instances in which teams translated observed patterns into recommendations. In one case, a team noted a higher prevalence of Rx dispensation for scheduled drugs among decedents in their county (56% vs 45% statewide), and identified a gap in what they described as a “transactional” system of care. They recommended educational initiatives and training for pharmacists to better support individuals in situations such as when medications are unavailable. In another instance, after observing a higher prevalence of jail bookings in their county (37% vs 25% statewide), a team recommended educating incarcerated individuals about changes in their drug tolerance following release.

Overall, dashboard use was rare during OFR meetings: teams used it only 10 times. Most of our 114 observations were *missed opportunities*, where teams did not consult available population-level dashboard data during discussions or recommendations about touchpoints in decedents’ cases. For example, in one meeting, the team reviewed a case where the decedent had a jail booking, 4 ED visits, and a prescription refill in the 12 months prior to their fatal overdose, yet the discussion centered on childhood factors and substance use disorder history. By contrast, the dashboard indicated that the county had a substantially elevated overdose rate following jail bookings (992 per 100,000 vs 469 statewide) and a slightly elevated Rx dispensation prevalence (40% vs 35% statewide). These data could have supported recommendations targeting more immediate intervention points, such as jails or pharmacies. In another meeting, despite a decedent having 18 ED visits and an EMS interaction in the prior year, the team focused on the decedent’s failure to use peer recovery programs rather than on the high-risk touchpoints themselves. The dashboard, by contrast, showed above-average county rates for both ED visits and EMS interactions and indicated that 25% of decedents with these touchpoints fatally overdosed within 2 weeks. These 2 examples illustrate missed opportunities to use dashboard data to identify and prioritize touchpoints based on their risk (rate), reach (prevalence), and immediacy (recency).

### Barriers to Integrating Population-Level Data in OFRs

#### Overview

We now discuss themes that emerged from interviews with OFR team members, including barriers to integration and future opportunities for data-driven prevention. Interviews shed light on several reasons behind the limited use of the dashboard during meetings. Reported barriers included tensions between case-based review and population-level analysis, varying levels of data literacy among team members, and limited time and resources.

#### Tension Between Case-Based Review and Population-Level Analysis

Participants highlighted a key distinction between OFRs, which focus on the detailed examination of a select few decedent cases, and the population-level data analysis provided by the dashboard. They contrasted OFRs’ case-specific, narrative-driven analysis with the broader, aggregate approach typical in academic and public health settings. This contrast implies that OFR members apply mental models and analytical processes that differ from how the data are presented in the dashboard:

*People we work with don’t think or talk about data in the same way, so we don’t think about what a quartile or an odds ratio is. At least not in the way it’s supposed to. And that’s some of the potential disconnect between something that is research–based and fact–based, and how we think of it in academia versus the random person on the street that will easily say, that’s 5 times more likely. And that’s not actually what they mean, but when it’s used in that academic setting, it can get a little bit lost in translation*.

[P1, nonprofit director]

Another aspect of the tension between approaches is the inherently empathetic nature of OFR case reviews, which is central to how teams understand cases. Team discussions focus on decedents’ lived experiences, using that perspective to identify the challenges they faced and the factors that may have contributed to their overdose. In some instances, team members even recognized the individuals being discussed or knew their families. This empathy and attention to individuals can feel at odds with the statistical abstraction of population-level data:

*What gives people motivation to work on top of their day-to-day workload on a goal is, their hearts, right? So, we just looked at the life and death of this individual we need to change X, Y, and Z, and these are the numbers to support it. I think that’s where data usually falls short. Because we were looking at this qualitative section of this life; details that the numbers are missing... that’s the SOFR [Suicide and Overdose Fatality Review] magic right there*.

[P5, MAT specialist]

#### Limited Data Literacy and Tacit Knowledge

All participants highlighted limited data literacy as a key barrier, making it difficult for them to confidently draw appropriate inferences from the dashboard. They pointed out that the OFR teams comprise members from diverse backgrounds, many of whom do not possess data analysis skills:

*Most of the room aren’t data people. They want to focus on the case, what happened, and how can we make this better for our county. When people ask for data, they want to know, but in a way that they can digest it. So, if we get too into numbers and aggregate, we lose people*.

[P5]

Participants noted that this limited experience raised a concern that the data could be misinterpreted, even with tooltip-style annotations and training videos included with the dashboard:

*We had a disagreement of what this actually means, and how do we actually interpret it? Because it has lots of data, that’s just really easy to get wrong*.

[P1, nonprofit director]

Participants also noted how interpreting the dashboard can be particularly challenging in small counties with lower populations:

*You have to really explain rate per 100,000... we’re working in rural areas where we have, 8 [overdoses] total in a year, but then we’re looking at numbers in the 50s and 60s, and so they’re like, wait, what’s happening? So, it isn’t something that someone could just pick up and understand*.

[P5, MAT specialist]

Participants also noted that many OFR members have a qualitative understanding of the key touchpoints in their community and do not feel the need to consult a dashboard to confirm their knowledge with raw numbers.

*We talk commonly in our meetings, [that] person was released from jail and died of an overdose 24 hours later. That’s common, we know that a lot of people do that, but we don’t put a specific number on it. We all see those issues happening every day, and we don’t need to say, 1 in 10 people getting released will experience this. Because of the work, we know the parameter without being able to put a specific number on it. There’s definitely value to know the actual statistics so we can keep educating the community and justifying the cause. But it just takes more time*.

[P7, PSW]

#### Perceived Data Stagnation and Insufficient Granularity

Although the dashboard updates in near real time, participants noted that its data rarely changed between meetings, as touchpoint prevalences and rates tend to remain stable over several months within most communities. This apparent stagnation appears to reduce the incentive to consult it regularly:

*We don’t need [the dashboard] all the time... ultimately, with this data, I doubt they’re changing drastically month to month. Once we’ve identified what our patterns are, that’s probably where it’s staying. And so, I think we use it when we need it, but when we need it is not a weekly or even usually a monthly thing*.

[P1, nonprofit director]

*It’s cumbersome to look at the dashboard at each meeting, because we’re not going to see a lot of change in the numbers. So, I try to space [usage] out [so teams] not looking at the same graph, the same numbers, the same data each time*.

[P5, MAT specialist]

One participant stated that they never used the dashboard because the data were not granular enough for their needs. While the dashboard breaks down touchpoints by county, consistent with how OFR teams are organized, larger urban counties tend to dedicate meetings that focus on risks for specific demographic groups or locales (eg, veterans, African American males over 50, overdoses at motels or hotels). This level of detail is not available in the dashboard:

*It [the dashboard] kind of presents some general ideas and directions for us to go, but typically our meeting themes are more granular and more subsect from the dashboard*.

[P4, Overdose Data to Action contractor]

#### Impact of Staff Turnover and Resource Constraints

Participants noted staff turnover and lack of resources as barriers to consistently incorporating the dashboard into meetings. They cited members leaving the team, facilitators having to manage multiple county meetings, and leadership and funding changes:

*We had built out a “here’s how we could use it, and here’s what we should use it for”. That got derailed when my staff person left. It needed somebody that wasn’t me to carry [the dashboard implementation] out... With some of the staff turnover, we have used it less*.

[P1, nonprofit director]

*IDOH covers the OFR teams, and there has been so much structural change over the last few years as far as leadership, it is difficult [for facilitators] to figure out who’s in charge, who do I go to [for directions].*

[P6, RN]

Participants also highlighted the high workload and the lack of time and resources as additional barriers:

*...people are pretty worn out. It was a hard time already doing all of this work, and now with all the funding cuts and the upheaval of nonprofits, they’re not quite sure which direction to go, so FORTRESS is a great dashboard, but sometimes it’s just one more thing [to learn and use]*.

[P6]

Importantly, one participant felt that dedicating a portion of the meeting to analyzing the dashboard data would, in effect, reduce the time available for case review, discussions, and/or recommendation making:

*I think one is time constraint. Do we do it at the beginning, middle, or have specific meetings? Every meeting feels different, the cases flow differently. It also depends on everyone’s vibe. We do two cases in two hours, and we are usually left with very little time to discuss recommendations*.

[P5, MAT specialist]

### Opportunities for Leveraging Population-Level Data in Prevention

#### Overview

Despite the obstacles to integrating the dashboard into OFR practices, participants highlighted several value points, both in and outside meetings, particularly in making local touchpoint data more accessible and serving as a resource for advocating policy changes.

#### Increasing Engagement Through Data

Participants noted that the dashboard can provide additional perspectives during meetings, particularly for touchpoints where representative stakeholders are absent:

*The biggest thing is who’s not at the table that can speak to that data intimately. We don’t have EMS services at our table, so when we’re going through recommendations, we don’t have that viewpoint. So, when we’re reflective on FORTRESS data, we can kind of talk to that*.

[P6, RN]

Conversely, it was noted that the dashboard can help identify prevention and improvement opportunities, especially when stakeholders who can understand the data and influence change at specific touchpoints are present at the meeting:

*If there are people in the room that have that ability to have impacts... having somebody in the room who can see the data, understand the data, and change something in my office. That’s where I think it’s [dashboard] most impactful*.

[P1, nonprofit director]

#### Access to Local, Timely Data

Even without full integration of the dashboard into OFR practices, team members recognized a key benefit: centralized access to comprehensive, timely, local-level data that may otherwise be difficult to obtain. Participants highlighted the value of the dashboard’s high-level county data, particularly for smaller counties with limited access to hospital and prison information:

*We’re in a smaller community... data that the FORTRESS dashboard uses isn’t usually data we have great access to like, Department of Corrections and EMS data. So it provides that guidance*.

[P1, nonprofit director]

Participants also cited the dashboard’s consistent updates as a major advantage (weekly for most data), even though they might not necessarily be needed during the meetings but for external needs such as pushing policies:

*What makes the dashboard really nice is that it is current data. I think a lot of boots on the ground in the community, or at least entities like us need up-to-date data at the drop of a hat for an XYZ reason*.

[P1, nonprofit director]

They noted the convenience of having all relevant data compiled in one place, explaining that this made it easier for teams to quickly spot trends and patterns that might have otherwise gone unnoticed:

*[Looking at] the prevalence, we were kind of like, whoa, I had no idea it was like this. So, we always hear about it, but to actually see the data, it was really eye-opening*.

[P3, behavioral analyst]

#### From Data to Action

Participants’ comments suggested that the dashboard has the potential to achieve its intended purpose. Three participants noted that it can help address limitations of the case-based review process by encouraging teams to consider how prevention opportunities might apply more broadly, beyond individual cases. As one participant explained:

*In our county, there are people coming out of prison, and we don’t have a lot of information, so I think that that would be a good opportunity to bring [the dashboard] up. What can we do, maybe not for this specific case, but for this population in general, how can we reach them?*

[P3]

Five participants noted that the dashboard can be a valuable tool for translating data into actionable insights, particularly for informing policy and programmatic decisions, although they recognized that realizing these opportunities often depends on external constraints, such as funding:

*The dashboard could really be useful if we get a state-level OFR partner group, where we were going to try to present things to policymakers, [eg,] being able to show we need to give a lot more support to our justice-involved individuals. That is a really clear takeaway from this dashboard... nice to have that kind of data there and visualized*.

[P4]

*When I was looking through the dashboard, I could think, we have 10 different initiatives that would be good at addressing what the data was saying. I think the question is always funding, right? So, we can look at the dashboard and say, I can envision XYZ, but we need the dollars to do it*.

[P7]

#### Other Dashboard Uses

Participants reported ways to use the dashboard that we did not observe directly, such as prioritizing recommendations:

*We had a recommendations meeting, that’s how we decide what recommendations to move forward on. We decided to focus on jail discharges, because that was huge... [and] this is a recommendation we can reach the most people with. I think it has been really impactful, because we had a lot of recommendations, and we were kind of like, where do we start?*

[P3, behavioral analyst]

Participants also reported use outside of OFR meetings to provide the data to support for certain recommendations and initiatives:

*I had pushed for having Narcan and test strips in our lobby and there was a lot of pushback against it. Through FORTRESS, [for] the first time I’ve been able to point to a statistic and say, ‘this is why it’s so important that we have this in the lobby.’*

[P7, PSW]

## Discussion

### Principal Findings

OFR teams are an increasingly important public health intervention for preventing overdoses through locally tailored strategies. Traditionally, these teams conduct case reviews for a small number of decedents to identify system gaps, risk factors, and corresponding prevention opportunities within their communities. The FORTRESS dashboard was designed to augment this case-based review process by incorporating population-level data on touchpoints that precede overdoses. This study sought to examine the extent to which teams integrated the dashboard into their workflows, identify barriers to its use, and highlight opportunities for more effective integration.

Over a 10-month period, we observed 11 OFR teams in 26 meetings and found limited integration of the dashboard into those meetings. Specifically, teams consulted the dashboard in only 8% (2/26) of instances where it could have informed a case discussion or recommendation. In those few instances, dashboard use ranged from simple data lookup to informing concrete recommendations, suggesting that it can support both basic and more advanced analytical needs. However, overall uptake was limited, as teams frequently discussed touchpoints for which the dashboard provided relevant data but did not appear to use it. In effect, we observed many more missed opportunities to incorporate population-level insights into teams’ discussions than actual uses. Interviews with team members uncovered several barriers to this limited uptake, as well as opportunities to improve integration.

### Barriers and Opportunities

Interviews suggest that OFR teams have a strong preference for traditional case-based discussion. The OFR review process is inherently qualitative, relying on narratives to reconstruct the decedents’ timelines and to understand key events, gaps, and factors that may have contributed to their overdoses. Teams also routinely draw on next-of-kin interviews, often attempting to interpret cases from the decedent’s perspective. This approach is deeply contextual, empathetic, and centered on individual lived experiences. Yet, this mode of sense-making can seem at odds with population-level analysis, in which individual experiences are abstracted into aggregate statistical measures. As a result, the case-based model for OFRs, while effective for generating contextual insights, may inherently discourage engagement with quantitative data, keeping teams focused on individual cases rather than broader risk patterns.

The current dashboard design, which separates population-level data from case-specific details, may have limited its use. Pisani et al [28] showed that data decoupling can increase OFR teams’ cognitive load and recommended integrating population-level data with case-level modeling tools within a single dashboard. Our findings support this approach but further indicate that visualizations should directly merge aggregate data with anonymized case details. For instance, a timeline view could show a decedent’s touchpoints while embedding population-level metrics within the same view. In such a design, individual touchpoints could be shown as points along a timeline, with their size reflecting prevalence among county residents (eg, larger points indicating more common occurrences) and color representing overdose risk (eg, deeper red indicating higher rates). By linking individual trajectories with broader population-level data in a single view, such visualizations could help teams better identify population-level patterns during discussions. This approach may also align better with existing OFR workflows, which focus on case narratives, making adoption more likely, as tools that match established practices are readily accepted [29].

Another consistently cited barrier to adoption is the limited data and visualization literacy among team members. OFR teams include stakeholders from diverse fields, such as social work, coroner services, and law enforcement, with varying levels of data expertise. Thus, difficulty in interpreting population-level data remains a key factor limiting dashboard use. These challenges are consistent with evaluations of public health dashboards, which indicate that users often struggle to interpret visualizations without support [13,30-32]. However, previous studies show that visualization skills can be effectively learned through video and interactive tutorials [33] and that users with low data literacy benefit from storytelling or narrative elements included in the dashboard [34]. In response, we developed additional training materials in the form of short videos that demonstrate how to interpret the dashboard and apply its data to make actionable recommendations.

Barriers appear to extend beyond data literacy. Some participants described the dashboard as an added burden alongside existing case-review responsibilities, leading to reluctance to engage with it. Others felt they already had sufficient knowledge of local risk factors and did not see the need to consult the dashboard, further limiting its uptake. One potential solution is to designate a dedicated data analyst within OFR teams to explore population-level data, interpret trends, and communicate key findings to the group. This approach allows other team members to focus on case discussion and recommendation-making rather than data interpretation [35,36]. Some teams that we observed have already adopted a similar model by appointing a “data specialist” to review external data sources. Traditionally, this role has involved working with tools such as REDCap databases, which provide limited quantitative information on reviewed cases. Expanding this role to include dashboard use, supported by additional training, could enable teams to better leverage it. This approach may help address both data literacy challenges and time constraints, possibly facilitating more effective integration.

A notable finding from our observations was the frequent missed opportunities to leverage the dashboard during meetings. Often, teams encountered touchpoints for which relevant data were available but did not consult the dashboard to inform their discussions or recommendations. This pattern indicates a need for more immediate support in identifying when and how to apply the dashboard. To address this, we began experimenting with a real-time feedback approach in which research team members observe OFR meetings and provide prompt, specific feedback afterward—highlighting missed opportunities and proposing ways to integrate dashboard data into the discussion. This approach builds on prior work showing that real-time feedback can help users overcome visualization literacy barriers [37] and that timely audit-and-feedback encourages change [38,39]. By clearly linking case discussions to relevant data features, this strategy may enhance teams’ use of population-level data.

While our observations showed limited uptake by OFR teams, we believe that providing near real-time data on overdose touchpoints can still be valuable to other localities or states. However, to increase the chance of use during meetings, the dashboard can benefit from several modifications, including innovative visualization designs that combine population-level data with case data, such that teams can more readily integrate the two into their review process. Another potential opportunity lies in the use of large language models to provide a more user-friendly chat interface for accessing and interpreting the data provided by the dashboard [40-42]. In particular, large language models can also provide a bridge between case narratives and aggregate data, helping team members leverage both analysis modalities to make actionable recommendations [43,44]. Additionally, improving training for OFR team members on data use and incentivizing evidence- and data-driven recommendations could encourage further adoption.

### Limitations

This study has several limitations that need to be considered. While we observed dashboard use during OFR meetings, we do not know how often or in what ways the dashboard is used outside those meetings. Therefore, we cannot fully determine how much it indirectly influences decision- and recommendation-making. Interview comments indeed suggest that the dashboard may have been used in other ways. Moreover, not all OFR members at the meeting were trained in dashboard use. Interviews suggest that some of the members who attended our 2-day training workshop may have left the team before our observations. Staff turnover could thus limit dashboard use during meetings. Additionally, only a subset of OFR teams in Indiana was observed, so the discussions and practices observed may not represent all OFR teams. Similarly, the interview findings were based on 7 participants, whose experiences and perspectives may differ from those of other OFR members not included in the study. The limited sample may have biased the themes identified in the study. Still, this study highlights a need for ongoing support and training to help OFR teams generate data-informed prevention strategies.

### Conclusions

This study examined whether OFR teams can effectively integrate a population-level touchpoints dashboard into their meetings to support data-driven recommendations. Our observations suggest that, while the dashboard can help teams generate useful insights and inform recommendations, its use during meetings was infrequent. Interviews with team members point to a tension between the case-based review model of OFRs and the population-level, data-driven approach afforded by the dashboard, with members struggling to incorporate the latter into their existing practices. Team members also cited limited data literacy, as well as time and resource constraints, as barriers to its use. These findings suggest a need to design data tools that better bridge the gap between case reviews and quantitative, epidemiologic analyses—for example, through visualizations that integrate individual case information with population-level metrics. Providing additional support and training for dedicated “data specialists” within teams may also help them leverage data and generate evidence-based recommendations, potentially leading to stronger prevention strategies at the local level.

## Supplementary material

10.2196/98864 Multimedia Appendix 1 Interview guide for semistructured interviews.

10.2196/98864 Checklist 1 COREQ checklist.

## Abbreviations

COREQ: Consolidated Criteria for Reporting Qualitative Research
ED: emergency department
EMS: emergency medical services
FORTRESS: Fatal Overdose Review Teams: Research to Enhance Surveillance System
IDOH: Indiana Department of Health
MAT: medication-assisted treatment
OFR: overdose fatality review
PSW: police social worker
RN: registered nurse
Rx: medical prescription
