# Supplementary material for: Barriers to Population Data Use in Overdose Fatality Reviews: Observational and Interview Study of Dashboard Deployment
Source: JMIR Hum Factors. 2026 Jul 27;13:e98864. doi: 10.2196/98864 (PMC13404934; doi:10.2196/98864)
Supplement: Multimedia Appendix 1 [file humanfactors-v13-e98864-s001.docx]

**Section 1:**

1. What is your background and role in the team?
2. Have you received any training or support in using data tools or dashboards before?
3. Were you aware of the FORTRESS dashboard before today’s meeting? How did you hear about it?
4. Have you accessed or looked at the dashboard prior to today’s meeting? What did you use it for and for how many times?

[If participant is not familiar with the dashboard, maybe provide a 2–3-minute intro/walkthrough to the dashboard at this point, and skip to section 3]

**Section 2:**

1. How easy is the dashboard to navigate and use?
2. How confident are you in your ability to interpret the data shown in the dashboard?
3. Do you feel the dashboard provided insights you wouldn’t have considered otherwise?

**Section 3:**

1. Have you ever used the dashboard to inform a recommendation during a case review?
   1. If yes, can you give an example?
   2. If not, do you envision using it in the future to make data-driven recommendations?
2. In what way do you think the dashboard can complement OFR case reviews?
3. What challenges – if any – will the team face in using the dashboard data during OFR meetings
4. How can the dashboard help teams make better recommendations?

**Section 4:**

1. What challenges do you face when using the dashboard in OFRs?
2. Are there parts of the dashboard that you find confusing or overwhelming?
3. What modifications would make it easier for you to use the dashboard more effectively?

**Extras:**

1. Which types of visualizations (e.g.: bar charts, line charts, maps) are easiest or hardest for you to interpret?
2. Is there anything else you’d like to share about your experience or needs related to data in the OFR process?
